# Multimodality imaging of an interventricular septum hydatid cyst

**DOI:** 10.1186/s43044-021-00147-8

**Published:** 2021-03-09

**Authors:** Krishna Prasad, Rupesh Kumar, Vikram Halder, Muni Raju, Sunder Lal Negi, Sanjeev Naganur

**Affiliations:** 1 Department of Cardiology, Advanced Cardiac Centre, PGIMER, Chandigarh, India; 2 Department of CVTS, Advanced Cardiac Centre, PGIMER, Chandigarh, India; 3 Department of Radiodiagnosis, PGIMER, Chandigarh, India; 4 Department of Cardiac Anesthesia, PGIMER, Chandigarh, India

**Keywords:** Hydatid cyst, Cardiac magnetic resonance imaging, Echocardiography, Computed tomography

## Abstract

**Background:**

Cardiac hydatid over the interventricular septum is extremely rare. Echinococcus infests humans as an accidental host. Echocardiography usually clinches the diagnosis of cardiac hydatid. However, multimodality imaging including cardiac magnetic resonance (CMR) imaging, computed tomography (CT), and positron emission tomography (PET) helps in supporting the diagnosis and surgical planning.

**Case presentation:**

We present a 29-year-old male who presented with dyspnea and was found to have cardiac hydatid on the interventricular septum on echocardiography. CT and CMR clinched the diagnosis. CT pulmonary angiography showed extensive pulmonary thromboembolization and cavitary consolidation in lungs. PET showed no active uptake in cardiac hydatid. Post-surgical enucleation of the cyst his hypotension worsened and succumbed.

**Conclusion:**

Cardiac hydatid has poor prognosis. Multimodality imaging helps in confirming the diagnosis and surgical planning.

## Background

Cardiac hydatid disease is very rare and its occurrence over the interventricular septum is even rarer. It is caused by infection due to Echinococcus granulosus which is still endemic in sheep breeding nations. Although echocardiography definitively identifies the disease, multimodality imaging including contrast enhanced computerized tomography (CT), cardiac magnetic resonance (CMR), and Positron emission tomography (PET) helps in delineating the lesion, defines the involvement of underlying structures, activity in the cyst and helps in defining the surgical fields. We present a case of cardiac hydatid disease on interventricular septum (IVS) along with liver parenchymal hydatid and secondary infection in the lung with pulmonary thromboembolism and underwent surgical enucleation of the cyst.

## Case presentation

**Fig. 1:**
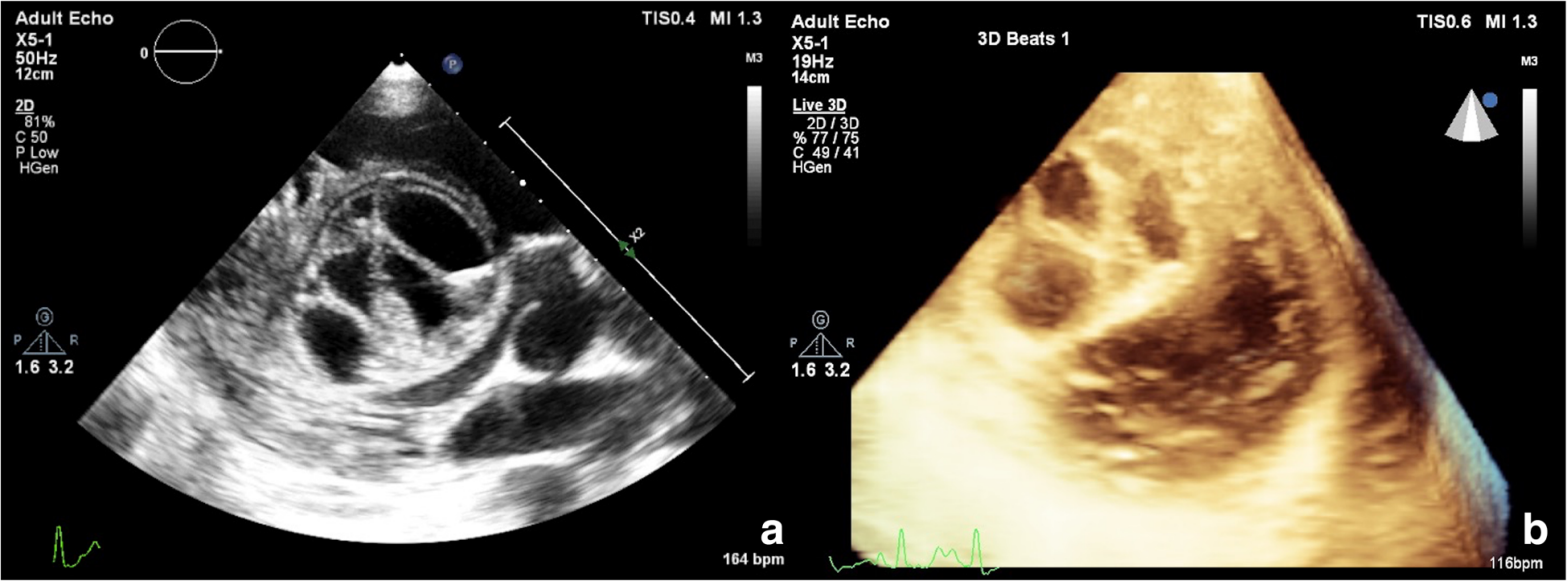
**a** 2D Echocardiographic and **b** 3D echocardiographic image showing a multilocular cystic mass over the interventricular septum

**Fig. 2:**
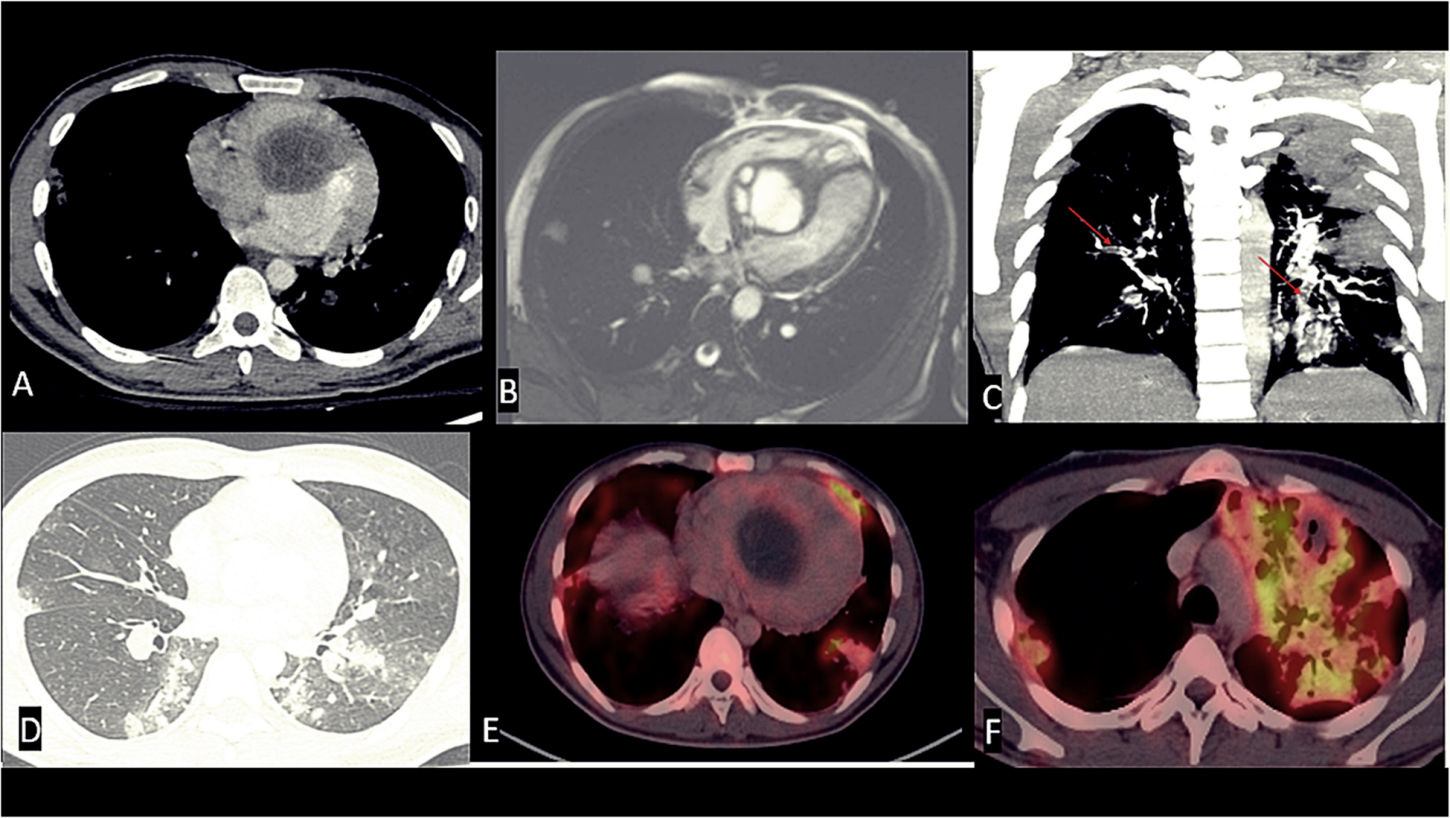
Contrast-enhanced cardiac CT (**a**), contrast-enhanced cardiac MR (**b**) axial images showing a well-defined, non-enhancing, uncalcified, multivesicular, multiloculated cyst (type II) at interventricular septum. Coronal reformatted CT images (**c**, **d**) showing segmental and subsegmental intra-pulmonary filling defects in bilateral pulmonary arteries; lung evaluation showing multiple, centri-acinar nodules with “tree in bud pattern.” PET CT showing (**e**) no activity at septal cystic mass (**f**) with avid uptake at left upper lobe consolidation

A 29-year-old male with no previous medical history presented to us with rapidly progressing dyspnea on exertion. He was admitted to the intensive care unit for further evaluation. His blood investigations showed anemia (hemoglobin 9.7 g/dl), normal renal, and liver function tests. His echocardiography revealed a large cystic mass in the right side of the IVS with hypokinesia of the involved septum and an ejection fraction (EF) of 35% (Fig. 1). For further delineation of the cystic mass a CMR was done which revealed a 4.5 × 4.5 cm well-defined hyperintense multilocular cystic lesion in the basal and mid IVS with reduced motion of involved septum and on post-contrast, there was less perfusion in the involved septum and a CMR calculated EF of 35% (Fig. 2a, b). Ultrasound abdomen also showed a calcified cystic lesion in the 7th segment of the liver. In view of multiple cystic masses a possibility of hydatid was kept, and he was started on albendazole 400 mg twice daily along with supplemental oxygen (FiO2 0.4 @ 8 l/min). His serology for hydatid was positive. As he was having continuous fever despite anti-parasitic therapy we have started broad spectrum antibiotics (piperacillin–tazobactam and vancomycin). Investigations done to look for any secondary infection were not helpful (blood culture was sterile, urine culture was sterile, procalcitonin was 0.4 ng/ml).

**Fig. 3:**
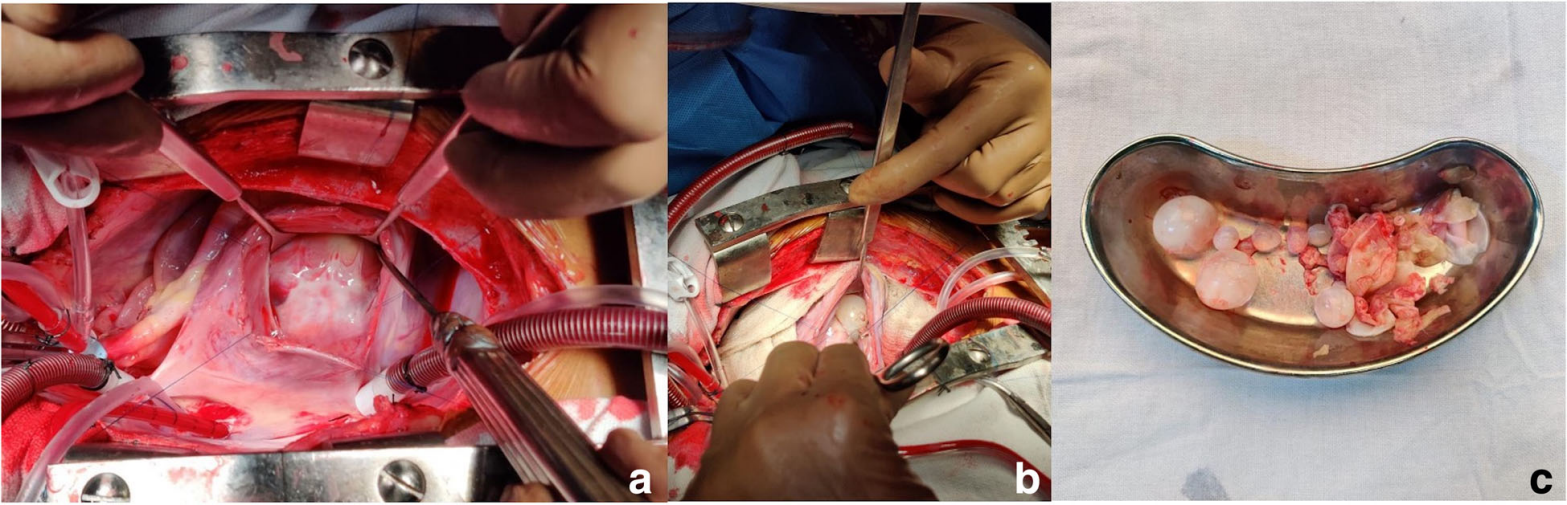
Surgical field images showing **a** Large hydatid cyst over the interventricular septum with intact wall. **b** Cyst wall was excised and daughter cysts inside the cavity can be seen. **c** Daughter cysts removed outside the patient

**Fig. 4:**
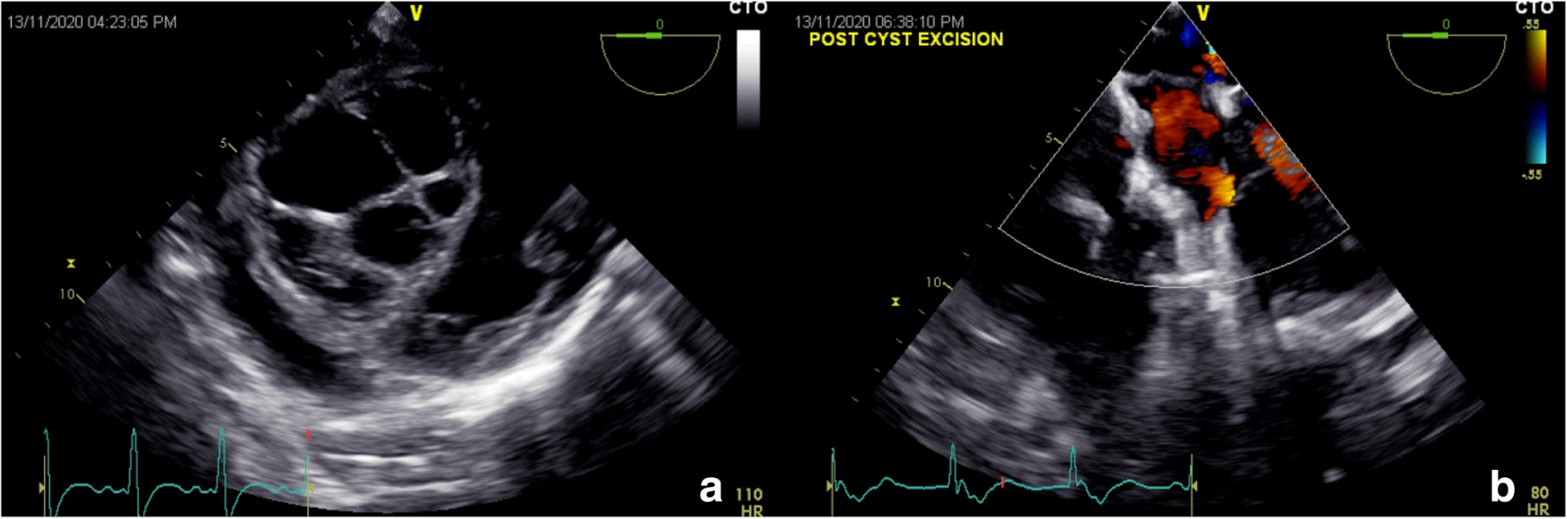
**a** Intraoperative transesophageal echocardiogram showing multilocular hydatid cyst over the interventricular septum. **b** Post-excision images showing complete removal of the cyst

Three days later, his dyspnea worsened with requirement of high amount of oxygen (FiO2 0.6, 15 l/min). Chest X-ray revealed multiple hazy opacities in bilateral lung fields. Contrast enhanced CT chest revealed multiple areas of cavitatory-consolidation and centrilobular nodules with tree in bud appearance and feeding vessel sign (Fig. 2d). CT pulmonary angiography showed a dilated main pulmonary artery (PA) and filling defects in the segmental and subsegmental PA’s (Fig. 2c). F^18^ Deoxy Glucose–Positron Emission tomography (FDG-PET) CT revealed low grade FDG uptake [Standardized uptake value (SUV) max 2.5] in the IVS and FDG avid (SUV max 4.6) multiple cystic lesions in the bilateral lung fields and one of them as a cavitatory lesion in the left upper lobe of the lung with intense FDG uptake (SUV max 10) (Fig. 2e, f). Owing to decrease in EF, large size of the cyst and with high risk of rupture, we went ahead with the surgical enucleation of the cyst. After midline sternotomy and heparinization aorto-bicaval cannulation was done. After application of aortic cross clamp and right atrium was opened and septal tricuspid leaflet was retracted. Incision was given in septum just below septal leaflet. Hydatid cyst was removed and septal incision was closed with 4-0 prolene (Fig. 3a–c). During cardio pulmonary bypass superior vena cava and inferior vena cava was snared to prevent inflow to the operative field. Right pulmonary artery and left pulmonary artery were also snared to prevent return. After excision of the cyst scolicidal agent 3% hypertonic saline was applied in right atrium and right ventricle and kept there for few minutes. After that right atrium and ventricle was washed with normal saline. After excision of the cyst all snares were removed. As both inflow and outflow were snared, dissemination of cyst and scolicidal agent has not occurred. After decannulation and insertion of chest drain and pacing wire, sternum was closed. Intraoperative transesophageal echocardiogram showed complete excision of the cyst (Fig. 4a, b).

Immediately post-surgery, the patient had difficulty in weaning from ventilation and point of care ultrasound showed high pulmonary arterial pressures. He developed refractory hypotension which could not be corrected even with the use of multiple inotropes and vasopressors (noradrenaline, dopamine, vasopressin). His antibiotics were changed to meropenem and vancomycin was continued. Despite the above measures his hypotension worsened and patient developed renal failure and ischemic hepatitis and disseminated intravascular coagulation. He succumbed 2 days later due to multiorgan dysfunction.

## Discussion

Cardiac hydatid cyst is a parasitic disease seen rarely in India. It is caused by infection with larvae of echinococcus granulosus. In the life cycle of echinococcus humans are the accidental hosts, dogs or other carnivores are the primary host and sheep as intermediate host. Humans get infected by eating food or fluids contaminated with dog feces containing ova of the echinococcus.

The most common site of the hydatidosis in humans is liver (50–70%), followed by lungs (5 to 30%), muscles (5%), bone (3%), kidneys (2%), spleen (1%), and brain (1%) [1]. Cardiac hydatid is uncommon occurring in less than 2% of cases [1]. Left ventricular free wall is the most common location followed by right ventricle, pericardium, left atrium, and right atrium. Interventricular septum is a rare location and occurs in less than 4% of cardiac hydatid cases [1]. Larvae reach the myocardium through systemic or pulmonary circulation or as a direct extension from other adjacent organs.

Clinical presentation depends on the location of the cysts, whether they are intact and infiltration of the underlying structures. Only few patients especially those with large size and involving underlying structures present with symptoms [2] like dyspnea, palpitations, angina, conduction disturbances, arrhythmias, valvular dysfunction, and outflow tract obstruction [3–5]. They can also present as systemic or pulmonary embolization [6]. Rupture of the cyst is a fatal complication resulting in anaphylactic shock, embolization, pericardial tamponade, and mortality in 75% of the patients. Very rarely, rupture may be silent. The cause for dyspnea and increase in pulmonary pressures in this patient was due to extensive involvement of lung due to pulmonary embolization.

Diagnosis is usually made by echocardiography and many times is sufficient [5]. Typical echocardiographic appearance includes well defined cystic mass with or without septations [7]. Echocardiography demonstrates the location of the cyst, its number and the relation with the surrounding structures, any valvular dysfunction, or outflow tract obstruction or pericardial effusion. In an echocardiography description study of 14 patients by Hamda et al., 8 patients had single cystic mass and 5 patients had multiple cysts [5]. A solid lesion can also be seen in some cases of infection with echinococcus multilocularis [2]. The differential diagnosis for a cystic mass on interventricular septum includes cardiac tumors, cysts, metastasis, IVS aneurysm, etc.

Further modalities like CT and CMR are very important in detailed characterization of the cyst, to assess the relation of the cyst with surrounding cardiac or extracardiac structures and helps in planning the surgery. The appearance of hydatid cyst on CMR is characteristic with hypointense on T1-weighted and hyperintense on T2-weighted. More specific signs include calcification and presence of daughter cysts [8]. CMR could identify additional cysts not identified by echocardiography [9]. Very few cases have been reported with nuclear imaging. There are no characteristic patterns on FDG PET for hydatid cyst and usually have peripheral calcification and minimal FDG uptake. However, very high uptake can mimic malignancy [10, 11]. In the present case, CMR was done to find out the nature of the cyst, its activity, and its relationship with the surrounding structures. With the use of CMR in our case, we could identify that the cyst is causing interference of contractility with hypokinesia of the septum, confirmation of decrease in EF, and also decreased perfusion in the septum was noted raising the possibility of infiltration of the underlying septum. The natural history of hydatid cyst is that the cyst grows slowly between the cardiac fibers and remain active for many years until it becomes large enough to cause compression, invasion of the surrounding structures [9].

Treatment involves combined medical and surgical therapy. Medical therapy includes albendazole which helps in preoperative sterilization of the cyst and decreases intraoperative dissemination. It is preferable to continue post-operative albendazole as it helps in reduction of recurrences [3].

Because of poor prognosis in case of rupture and only partially effective medical therapy, preferably all cases of cardiac hydatid cyst should undergo surgical excision. Surgical techniques depend on the location of the cyst and most often done under cardiopulmonary bypass. One of the problems during surgery is the extreme fragility of the cystic wall and risk of dissemination into systemic circulation. To prevent this, before enucleation an anti-helminthic solution (like 2% formalin, 0.5% silver nitrate solution, 20% hypertonic saline solution, 1% iodine solution, or 5% cetrimonium bromide solution) is to be injected in to the cyst [2].

In our case, as the patient was running fever despite multiple antibiotics and anti-parasitic therapy, we have done PET which showed no uptake in the cardiac hydatid cyst. We went ahead with surgery in this patient even though PET showed less FDG uptake as the cyst was affecting left ventricular contractility and because of the risk of rupture. This case highlights the poor prognosis of cardiac hydatid cysts, associated complications, and underlines the importance of early recognition and surgical management in such patients.

## Data Availability

The datasets used and/or analysed during the current study are available from the corresponding author on reasonable request.

## Abbreviations

CT: Computed tomography
CMR: Cardiac magnetic resonance
PET: Positron emission tomography
FDG: Fluoro deoxyglucose
IVS: Interventricular septum
SUV: Standardized uptake value
